# Closed reduction of Pilon fractures using an ankle distractor to allow for minimally invasive fixation

**DOI:** 10.1308/003588412X13373405387096j

**Published:** 2012-05

**Authors:** MSL Webb, P Bansal

**Affiliations:** Sunderland Royal Hospital,UK

The soft tissue injury associated with Pilon fractures is significant. Fixation using minimally invasive techniques allows for early ankle mobilisation while protecting the soft tissue envelope. Ligamentotaxis using an ankle distractor is a non-invasive reduction technique that protects the soft tissue and maintains access for minimally invasive fixation. The patient is positioned supine with a bolster under the knee. The sterile Guhl Non-Invasive Ankle Distractor (Smith & Nephew Orthopaedics Ltd, Warwick, UK) is applied and attached to the table using an extension bar under the drapes. Traction is applied. An upturned bowl under the calcaneus avoids sag.

**Figure 1:**
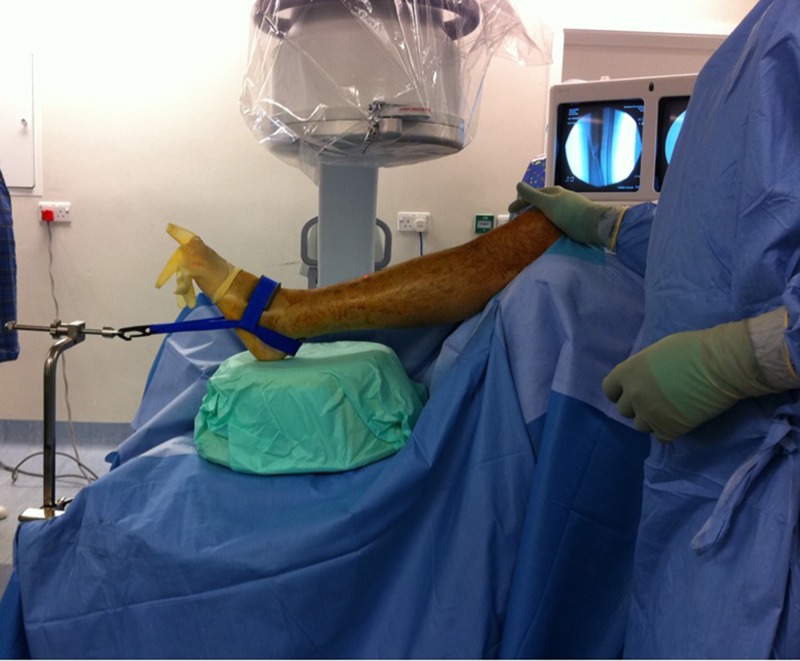
Image demonstrates set-up

